# Manual vs Mechanical Oral Hygiene Procedures: Has the Role of the Dental Hygienist in Phase 2 Post-lockdown Really Changed?

**DOI:** 10.3290/j.ohpd.b871059

**Published:** 2020-12-14

**Authors:** Elisabetta Polizzi, Giulia Tetè

**Affiliations:** a DH, Chair of Center for Oral Hygiene and Prevention, Department of Dentistry, IRCCS San Raffaele Hospital, Vita-Salute University, Milan, Italy. Idea, performed analysis, revision and final review of the manuscript.; b Dentist, Department of Dentistry, IRCCS San Raffaele Hospital, Vita-Salute University, Milan, Italy. Collected data, performed analysis, wrote the manuscript.

**Keywords:** Covid-19, manual hygiene, mechanical procedures, post-lockdown, role of hygienist

## Abstract

**Purpose:**

Some authors have highlighted the danger of intraoral use of rotating instruments which can produce droplets and aerosols. During the Covid lockdown phase, dental operators were limited to providing emergency treatment that could not be postponed. Therefore, it is necessary for the dental team to restart safely to treat neglected oral diseases that may also affect systemic health. The role of the dental hygienist has apparently changed for procedures performed close to the patient’s oral cavity, particularly in terms of the droplets and aerosols produced during oral hygiene practices. Through an analysis of the most recent literature on the use of dedicated PPE and changed post-Covid 19 work processes, and a review of the differences between manual causal therapy and mechanical therapy in terms of outcome after oral hygiene treatment, we define how the role of the dental hygienist can change positively.

**Materials and Methods:**

Narrative reviews of the literature in terms of PPE adopted and oral hygiene procedures performed were carried out in Pubmed.

**Results:**

188 articles from February 2020 to May 2020 using the search terms ‘dentistry’ and ‘covid-19’ were examined. 10 reviews of the literature were performed using the search terms ‘mechanical procedures’ and ‘manual hygiene’.

**Conclusion:**

Only continuous update of evidence-based literature on the new standards in oral hygiene procedures and the different results yielded by different procedures can ensure a safe working environment for the dental hygienist while supporting the dentist in this phase of the pandemic.

A novel corona virus, SARS-COV-2, arose in Wuhan, China in December 2019. In Italy, it spread first in Lombardy in February 2020, and then all over the world. The WHO declared a pandemic; the number of infected and mortality cases around the world fluctuates around 8%.^34^

The disease is caused by a new member of the Coronaviridae family, which the world already encountered as Severe Acute Respiratory Syndrome (SARS) in 2003 and Middle East Respiratory Syndrome (MERS) in 2011.^12^Studies carried out on these highlighted their pandemic and epidemic potential and their ability to transmit from animal to humans.^40^A study in New England reported that previous SARS and MERS viruses remain on surfaces for more than 72 h.^49^

The origin of the novel coronavirus SARS-COV-2 is still uncertain; however, the initial transmission appears to have occurred from animals to humans at the Wuhan market in China in December 2019, causing severe respiratory disease with cluster pneumonia.^34^

The virus generates a human-to-human transmissible parainfluenza syndrome, characterised by mild symptoms such as cough, chills, headache, and fever, which however can progress to pneumonia with severe respiratory conditions, sometimes associated with Acute Respiratory Distress Syndrome (ARDS).^27^ Sars-CoV2 can be transmitted directly via droplets (such as sneezing or coughing), direct contact, and contaminated material.^47^ The incubation period for individuals infected with 2019-nCov ranges from 1 to 14 days (on average 8 days), and individuals who first showed symptoms after 24 days of infection have also been reported.^20^ Furthermore, there is currently no scientific evidence in the literature regarding the availability of validated and effective rapid serological tests for the diagnosis of positivity or presence of immunoglobulins for Sars-CoV-2.

Dental professionals are exposed to infections daily, due to proximity to the oral cavity, and depending on the type and frequency of dental procedures.^19^ Some authors have highlighted the danger of intraoral use of rotating instruments, which can produce aerosols.^3^

During the lockdown phase, dental operators were limited to guaranteeing emergency treatment. Therefore, it is now necessary that dental team restart safely to treat neglected oral diseases that may also endanger systemic health.

The dental hygienist must carry out prophylactic treatment in patients to promote or maintain their oral health; moreover, the hygienist can motivate the patient to regain confidence in oral health care. However, some authors highlight that the lockdown led to a neglect and thus aggravation of oral pathologies. If neglected, an acute infection in a patient can lead to the formation of an abscess, and failure to check the oral cavity can mean neglecting trauma or injury in the patient.^54^

Other authors have pointed out how psychological stress of healthcare workers is increased during states of emergency. For instance, a questionnaire sent to Israeli dentists and hygienists showed an increase in psychological suffering, stress overload, and phobic pathologies regarding the transmission of the new SARS-COV-2.^28^

Our article aims primarily to report all the safety protocols for oral hygiene procedures in terms of reorganising the waiting room and reception area, PPE (Personal Protective Equipment), management of activities, environmental disinfection, sanitation and reorganisation of work, while also considering the devices that technology has made available during this period. Furthermore, a review of the literature on the significant differences between manual vs mechanical oral hygiene procedures was conducted.

This can motivate dental hygienists and other healthcare workers to work safely by restarting together to ensure the patient’s oral health.

## Materials and Methods

### Operational Protocols for Oral Hygiene Procedures

From a review of 188 articles (40 were discarded as dentally irrelevant and 10 due to lack of full text), we formulated a protocol valid for both the dental hygienist and the dentist. We perform a double triage:^1^ by telephone the day before the patient’s visit and then at the office, so that a double check can be carried out at different times to confirm the patient’s health, identify those cases defined as fragile, and provide help for the National Health System.^52^

At triage, the patient places all her/his personal effects in a special plastic bag, wears a surgical mask, and washes her/his hands with a hydroalcoholic solution provided in appropriate dispensers.^10^ Before the patient’s arrival, the dental unit, which is considered a high-risk area, must be ready. Therefore, the surfaces must be free of objects, the keyboards must be covered with polyethylene to allow daily cleaning, and the instrument kit must be ready and covered until the patient arrives.^15^

Before carrying out any type of procedure, the patient must perform two rinses. The first rinse has a virucidal action with a 1% solution of hydrogen peroxide (one part 10-volume hydrogen peroxide 3% and two parts water) or gargling for 30 s with 1% povidone-iodine or with CPC (cetylpyridinium chloride) at 0.05–0.1% for 1 min. The second rinse serves to reduce the bacterial load with 0.2–0.3% chlorhexidine mouthwash for 1 min.^7^,^35^

If the reason for the patient’s attendance is an initial visit or a follow-up then s/he only wears the disposable water-repellent cape, possibly without a chain, but with laces to minimise the risk of contamination.

**Fig 1:**
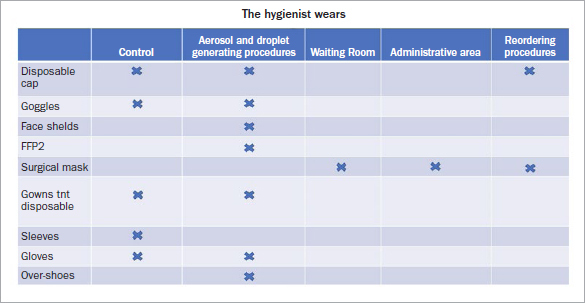
PPE worn by the oral hygienist in all areas of the dental practice.

The operational indications from the Italian Ministry of Health define initial visits and control/check-up procedures as medium-high risk, since the Ministry has recommended a higher level of safety in this phase. This may eventually be mitigated in a future phase 3 also for health workers. Therefore, the operator wears disposable TNT caps, protective goggles that must be placed over any eyeglasses, FFP2 facial respirator, TNT disposable or reusable gown, disposable sleeves (to reduce the turnover of white coats) and gloves (Fig 1).^35^

**Fig 2:**
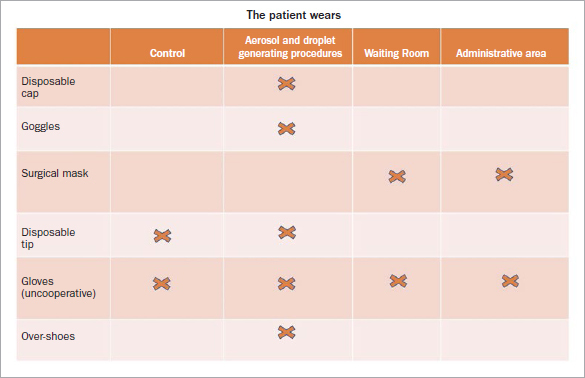
PPE that the patient must wear in areas of the dental practice.

If, on the other hand, the patient must undergo a procedure that involves the formation of aerosols, droplets, and biological liquids, the risk is defined as high. Thus, s/he must wear disposable TNT cap, protective glasses, disposable water-repellent cape, and shoe covers (advisable).^35^In this situation, the operator must wear a disposable TNT cap, protective glasses placed over any eyeglasses, face shield, FFP2 respirator, disposable TNT gown or reusable in TTR or TTR suit, and gloves (Fig 2).^35^Remembering that no matter what PPE we wear, it is always advisable to wash hands with soap and water for 40–60 s and/or hydroalcoholic solution for 20–40 s. Extreme attention must be paid to the undressing procedures since our PPE is contaminated.

Since these are high-risk procedures, the use of new devices is recommended, such as high-speed vacuums, which technology has made available to the operator to ensure safety and shorten working times.^44^ If the patient is a carrier, it is preferable not to let them enter, based on the degree of collaboration with the patient. If the patient is not compliant, the dental professional can enter and must be managed like the patient, to minimise the risk of contaminating the environment outside the dental unit, the other operators, and the subsequent patient as well as other persons present outside the dental unit.

Where it is possible in oral hygiene as in other dental procedures, manual procedures should be preferred over mechanical ones, and if handpieces are used, they must be used at low RPMs.^29^

### Manual vs Mechanical Procedures

A review of the literature was performed in the Cochrane Library and on PubMed (PCM) using the keywords sonic/ultrasonic scaling and root planing, as well as manual scaling and root planing. It is interesting that more articles were published in the past; comparatively fewer have been published recently, and the topic seems neglected. In fact, the bibliography mainly concentrates on the 1980s and 1990s. At a time like this, it would be useful to understand whether the same results can be obtained in terms of reduction of PPD, BOP (clinical indices), and invasiveness using manual vs mechanical hygiene procedures. Publications on case reports which had limited case histories and concerned patients with pre-existing systemic diseases were excluded.

## Results

Twenty articles were found, and of these, 10 articles were considered valid due to the presence of full text, relevant topic, number of the sample taken into consideration and corresponding result percentages.

The main observation on this issue is that there is no suitable scientific method to predict the maintenance of a dental element with periodontal disease by performing nonsurgical therapies, although analysing the reduction of attachment loss can be predictive for a result hypothesis.^52^ In the first study considered, the authors emphasised that in terms of clinical parameters, there is no strong evidence favouring mechanical or manual debridement of the roots; however, they argue that sonic and ultrasonic mechanical therapies require less time than manual treatment, producing less discomfort in the patient.^22^

An interesting in vitro study^38^ was conducted on 20 extracted teeth. The root surfaces were used to test the difference between mechanical tips and manual curettes in terms of tartar removal and post-treatment uniformity of the root surface. Neither group found statistically significant values with no differences between the two techniques used in terms of reduction of residual tartar. However, in terms of post-treatment surface uniformity, the manual curettes left the root surface smoother and more uniform than did the mechanical procedures.^38^

A similar study^6^ was conducted in vivo on 30 non-molar teeth with periodontal probing depths between 4 and 7 mm, already assigned for extraction for other dental reasons, using manual curettes and ultrasonic tips. The teeth were instrumented with the two techniques and then extracted to evaluate the amount of tartar removed. Once extracted, the teeth were stained with toluidine blue and examined with a scanning electron microscope (SEM). The results showed that the residual bacterial colonies after both types of instrumentation were minimal; thus there was no statistically significant difference between the two procedures. However, the fact that there were fibrin residues was highlighted.^6^

Another in vivo study on mechanical and manual instrumentation of the dental elements treated in flap surgery, which were subsequently extracted and examined under a microscope, showed a different result than in the previous papers. With manual instrumentation there was a lower percentage of tartar residues (5.8%) compared to teeth treated with ultrasound (6.2%).^21^

An interesting review from 2016^25^ found no statistically significant difference between manual and ultrasonic therapy either in terms of the supragingival or subgingival results. Rather, the two different treatment types used in combination yielded an optimal result in terms of improving clinical parameters.^25^

Some authors claim that manual therapy removes more dental tissue than does mechanical.^48^ Moreover, manual instruments must be periodically sharpened to optimise the smoothing of the dental roots.^4^ Some authors suggest sharpening curettes after a certain number of strokes; some suggest doing so after 5 strokes with a single curette,^33^ while recommend sharpening after 10-12 strokes.^13^,^39^ The literature shows that very few dental hygienists sharpen their curettes after 5-20 strokes.^53^ To address this problem, several studies focused the introduction of new metal alloys, including stainless steel, high-speed steel, carbon mixed with steel and tungsten carbide, which were shown to influence the effectiveness and service life of the instrument.^45^

Ultrasonic instruments need irrigation; the recommended flow rate is at least 20-30 ml/min to avoid a temperature increase over 5°C, which could damage the pulp and dentin.^31^ The different ultrasound inserts have a high variability because specific power levels are required for each of them to produce the same result in terms of plaque reduction.^26^

**Fig 3:**
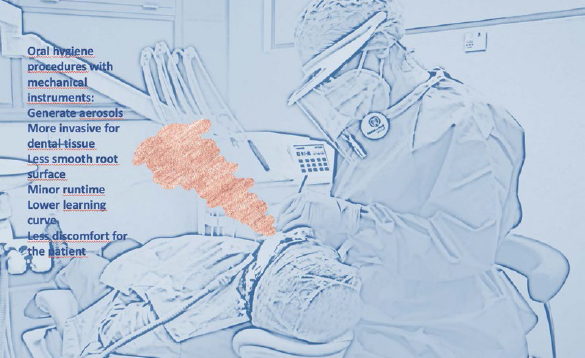
Oral hygienist during the mechanical procedures; the pink smoke symbolises the aerosol generated by the instruments. The advantages and disadvantages of mechanical therapy are summarised.

**Fig 4:**
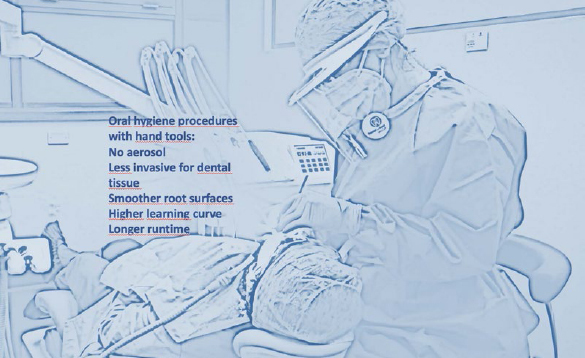
Hygienist during manual oral hygiene procedures; the advantages and disadvantages of manual technique are summarised.

An interesting review by Needleman et al^30^ shows no statistically significant difference between oral hygiene techniques performed with manual and mechanical instruments (Figs 3 and 4); however, the use of curettes produced smoother root surfaces than did the mechanical instruments.^30^ Some authors even report improvements in clinical indices and smoother surfaces with the use of manual curettes, but with a greater loss of tooth substance.^5^

Another of the objectives of root planing is to promote the adhesion of fibroblasts to the root surface. Several studies have shown that only a small number of fibroblasts attach to periodontally diseased root surfaces.^2^

With the use of diamond curettes, better-organized fibroblasts were seen on the root surface, which favours moderate roughness.^14^ However, it has been shown that bacteria could be attracted to a rougher surface, so more scientific study should be done.^24^ Technology has made new-generation ultrasound instruments available with more effective action than their predecessors, but results are in any case comparable to those obtained with manual instruments.^43^

## Discussion

Our analysis of the current literature shows that manual and mechanical oral hygiene therapies achieve the same results in terms of improving clinical indices. However, this study is limited by the fact that few articles have been published on manual vs mechanical treatment.

This is essential, because at a crucial moment such as the restart after Covid-19 phase 2 post-lockdown, the dental hygienist must be able to go back to work safely, avoiding the problems of aerosol. From our first analysis, we formulated a protocol to allow the hygienist to work safely.

During this period, patients only sought dental treatment for emergency services that could not be postponed, neglecting other pathologies within the oral cavity. Due to the potential danger inherent in Covid-19 and the current absence of a cure or vaccine,^16^some authors continue to recommend postponing dental treatment to reduce SARS-COV-2 transmission risk, instead preferring telemedicine.^36^

Given our clinical practice, in our opinion no patient should be put off, for several reasons. For example, patients who have poor oral hygiene risk developing more serious diseases, at the local and systemic levels, since they rely heavily on professional prophylaxis to improve oral hygiene.^46^ Some authors point out that professional prophylaxis is necessary in the uncooperative patient to maintain proper oral hygiene and therefore safeguard the patient’s health.^41^

Other authors underline the importance of the hygienist is in the follow-up of complex rehabilitation. In our hospital, for example, the external part of the cranial theca is removed for use as an autologous bone graft when the patient has severe bone atrophy, which cannot be resolved with other techniques. Such cases require constant monitoring for the first few months and maintenance of oral hygiene.42-50

Patients with autoimmune diseases, who we normally treat with implant-prosthetic rehabilitations at Vita-Salute San Raffaele Hospital, must have physiological and constantly monitored periodontal clinical indices, as they have a greater risk of implant loss than patients without autoimmune diseases.^8^

Patients who have undergone extractions need constant wound control, in which bone remodeling takes place after healing, and a good level of oral hygiene to prevent infection of the alveolus.^11^

Other authors emphasise that a prosthesis which creates occlusal problems can be a psychosocial problem for the patient, causing discomfort in vital functions.^9^

It is also necessary to mention all those patients sent to the oral pathologist for precancerous lesions detected during oral hygiene sessions; had these lesions not been detected in time, the patient’s health would be seriously compromised.^17^

In an epidemiological study^18^ conducted at the Changsha Stomatological Hospital in China from January 23 to March 2, 2020, 3500 patients received dental treatment. With the appropriate PPE, no infections were found in the operators or the patients.^18^

Supporting these statements and our work, Jongbloed-Zoet^23^ highlighted the fundamental role of the dental hygienist in preventing diseases of the oral cavity, which affect more than 3.5 billion people in the world, and in motivating patients to comply with oral hygiene measures to prevent these diseases.

In December 2019, the European Parliament stated that dental caries, if left untreated, is the most common non-communicable disease worldwide, incurring a total cost of an estimated at €100 billion annually for oral procedures in the EU. They estimated that productivity losses due to dental diseases reach around €57 billion per year.^37^

However, some authors still speak of an emergency, due to the high risk of exposure by the dentist or dental hygienist to the new SAR-COV-2.^51^

From clinical experience at our hospital during the pandemic emergency and from scientific evidence, we can say that dental team must be able to work safely, respecting all the operational safety indications, with particular attention paid to the appropriate PPE, the reorganization of work paths and workflows, as well as management of common areas, disinfection and sterilization processes. The message we want to send to patients today is that with the help of clear rules and very strictly observing them, we can go back to dealing with patients’ oral health. The patient must be able to resume trusting dental professionals, wjp hold patient health as the absolute prerogative. In this context, the dental hygienist plays a fundamental role, because thanks to her/his empathic relationship with the patient, s/he can convince patients of the safety of all dental procedures.

## Conclusion

In phase 2 (post-lockdown) of Covid-19, many papers have stressed the high risk of infection for hygienists. However, none stressed how important it is to restart non-emergency treatment in order to not further neglect oral disease, nor how important it is to build patient loyalty and motivation to perform dental care. The literature contains some safety protocols that lack clinical evidence. The present study describes implementing the operational safety instructions in practice by recommending the appropriate PPE to perform oral hygiene measures. The present article also reviewed the literature, which clearly highlights the potential of a manual oral hygiene considered ‘safe’ by most authors, compared to the mechanical procedure which generates aerosol.
